# Optimal water supply reservoir operation by leveraging the meta-heuristic Harris Hawks algorithms and opposite based learning technique

**DOI:** 10.1038/s41598-023-33801-z

**Published:** 2023-04-28

**Authors:** V. Lai, Y. F. Huang, C. H. Koo, Ali Najah Ahmed, Mohsen Sherif, Ahmed El-Shafie

**Affiliations:** 1 Department of Civil Engineering, Lee Kong Chian Faculty of Engineering and Science, Universiti Tunku Abdul Rahman, Kajang, Selangor Malaysia; 2 Institute of Energy Infrastructure and Department of Civil Engineering, College of Engineering, Universiti Tenaga Nasional (UNITEN), 43000 Selangor, Malaysia; 3 Civil and Environmental Engineering Department, College of Engineering, United Arab Emirates University, P.O. Box 15551 Al Ain, United Arab Emirates; 4 National Water and Energy Center, United Arab Emirates University, P.O. Box 15551, Al Ain, United Arab Emirates; 5 Department of Civil Engineering, Faculty of Engineering, University of Malaya (UM), 50603 Kuala Lumpur, Malaysia

**Keywords:** Climate sciences, Engineering

## Abstract

To ease water scarcity, dynamic programming, stochastic dynamic programming, and heuristic algorithms have been applied to solve problem matters related to water resources. Development, operation, and management are vital in a reservoir operating policy, especially when the reservoir serves a complex objective. In this study, an attempt via metaheuristic algorithms, namely the Harris Hawks Optimisation (HHO) Algorithm and the Opposite Based Learning of HHO (OBL-HHO) are made to minimise the water deficit as well as mitigate floods at downstream of the Klang Gate Dam (KGD). Due to trade-offs between water supply and flood management, the HHO and OBL-HHO models have configurable thresholds to optimise the KGD reservoir operation. To determine the efficacy of the HHO and OBL-HHO in reservoir optimisation, reliability, vulnerability, and resilience are risk measures evaluated. If inflow categories are omitted, the OBL-HHO meets 71.49% of demand compared to 54.83% for the standalone HHO. The HHO proved superior to OBL-HHO in satisfying demand during medium inflows, achieving 38.60% compared to 20.61%, even though the HHO may have experienced water loss at the end of the storage level. The HHO is still a promising method, as proven by its reliability and resilience indices compared to other published heuristic algorithms: at 62.50% and 1.56, respectively. The Artificial Bee Colony (ABC) outcomes satisfied demand at 61.36%, 59.47% with the Particle Swarm Optimisation (PSO), 55.68% with the real-coded Genetic Algorithm (GA), and 23.5 percent with the binary GA. For resilience, the ABC scored 0.16, PSO scored 0.15, and real coded GA scored 0.14 whilst the binary-GA has the worst failure recovery algorithm with 0.09.

## Introduction

Growing water demand would strain most reservoir storage systems. Optimising reservoir activities can increase water storage to alleviate this issue. More recent review in reservoir optimisation can be found in Ref.^1^. Furthermore, the reservoir optimisation using machine learning approaches was anticipated^2,3^, beginning with linear optimisation, then dynamic optimisation, and eventually heuristic algorithms, especially the Genetic Algorithms (GA). However, the authors of this study assert that these algorithms have their constraints due to the convergence speed and curse of dimensionality. To address these shortcomings of the previous methods, meta-heuristics algorithms (MHAs) were implemented for optimal reservoir operation in terms of standard operating system^4–6^ and management to solve the optimal nonlinear, nonconvex relationships and complicated problems with difficult and complicated thresholds^7^. The following paragraph mentioned a few of the recent MHAs been utilised in the different reservoir operation functions.

The implementation of reviews, as well as their disadvantages and benefits with different MHAs to optimise hydropower energy production has been highlighted in Ref.^8^. The adoption of the evolutionary algorithm in resolving the reservoir optimisation operation has been demonstrated in Ref.^9^. Meanwhile, the hybridisation of coral reefs algorithm and machine learning to solve the multi-reservoir system operation has been presented in Ref.^10^. From 2010 to 2019, the investigations of the time series data for optimising hydropower activity at the Karun-4 reservoir in Iran has been conducted in the studied found in Ref.^11^. This study demonstrated the evolutionary algorithm of GA, swarm-based algorithms of particle swarm optimisation (PSO), and a new nature-inspired swarm-based algorithm, the month swarm algorithm (MSA), used in reservoir hydropower optimisation and the reservoir simulation outcomes showed that the MSA algorithm excelled over the GA and PSO algorithms. Further on, the comparison of the operation management of the single hydropower reservoir between the Jaya Algorithms and other MHAs based on the hedging policy has been demonstrated in Ref.^12^. Although the Jaya algorithm had outperformed other MHAs in terms of achieving better solutions, it loses out in terms of convergence speed when compared to the PSO. Regardless of what has been interpreted above, as stated in the No Free Lunch Theory (NFL), as in any optimisation issue, there is no algorithm that can perfectly measure up to all of it^13^].

As such, more work is needed to prove the efficacy of the Harris Hawks Optimisation (HHO) and the Opposite Based Learning (OBL-HHO) algorithms and these algorithms have yet to be applied at the Klang Gate Dam (KGD), even though the HHO has been applied in reservoir irrigation management which can be found in Ref.^14^. Hence, this paper is an extension of the whale optimisation algorithm and the Lévy flight distribution conducted at the KGD by Ref.^15^. The following section describes the HHO algorithm, OBL-HHO, study area, and dataset. The study flowchart is also presented. Optimal KGD release curves have also been plotted. The suggested MHAs are compared to other published heuristic algorithms in terms of algorithm performance and reservoir risk analysis. Statistical errors were also evaluated. This work optimised the KGD operations with multi-objectives by utilising the HHO algorithm and OBL-HHO approaches to minimise water deficit. Lastly, the novelty of this work has been highlighted in earlier statement of this paragraph and the results have been compared to other previous heuristic algorithms in terms of reservoir risk analysis, in order for the stakeholder to consider which criteria of the reservoir risk analysis to be selected, correlated to the algorithms without jeopardising the operations of the KGD and downstream activities.

## Methodology

### Study area and datasets

#### Study area

The Klang Gate Dam, often known as the KGD, is located in the Taman Melawati neighbourhood of Kuala Lumpur's Gombak district. It is also known as the Bukit Tabur Dam, and the numbers 3217002 and 3217004 belong to the rainfall stations located on site (Source: Department of Irrigation and Drainage, Malaysia). The dam was constructed at a latitude of 3° 13′ 58″ North (3.233°) and a longitude of 101° 45′ 0″ East (101.75°). The characteristics of the KGD is outlined in Ref.^16^, and its major objective is to deliver water to the two water treatment plants (WTPs) located at Wangsa Maju and Bukit Nanas districts in the Kuala Lumpur City region.

#### Datasets

Observed monthly Storage, inflow, evaporation rate, and release data were collected for the years 2001 to 2019 *[Source: Lembaga Urus Air Selangor (LUAS) or Selangor Water Management Authority].* The inflow was classified into three categories, namely high, medium, and low flows, which can be found in Ref.^15^. According to Ref.^15^, Puncak Niaga (M) Sdn Bhd is the company that is involved with the running of the dam operations. The HHO and OBL-HHO were utilised to optimise the monthly reservoir release operation at the KGD. This was accomplished with reference to the monthly observed dataset of inflow, demand, storage, and losses (measured in a million cubic meters, MCM). The optimisation process with the datasets mentioned, was performed with the Python (Anaconda) software.

### Formulation of the optimal release from the reservoir

#### Objective function

- Minimisation of water deficit.
  The objective of this function is to bring the monthly water deficit down to the minimum value, whereby *Z* described as the deviation between water demand and water release, as defined by Eq. (1).
  1 $$Min\;Z={\sum }_{t=1}^{12}{{(D}_{t}-{R}_{t})}^{2}$$
  where *t* represents the number of months in a year, and *D*_*t*_ and *R*_*t*_ represent the monthly demand and release for that month, respectively.

#### Thresholds

In order to achieve optimal reservoir operations, every reservoir system must adhere to the constraints and penalty functions. The upper and lower boundary limits of the static penalty function, also known as the thresholds, were used to achieve optimum reservoir operation without jeopardising the objective function^17^.

- Equality of continuity threshold.
  Equation (2) gives the Continuity threshold:
  2 $${S}_{t+1}= {S}_{t}+{I}_{t}-{R}_{t}-{L}_{t}$$
  in which ${S}_{t+1}$ and ${S}_{t}$ are the final and initial storages, for time *t* (monthly), respectively; ${I}_{t}$ represents the inflow to the reservoir; ${R}_{t}$ represents the reservoir’s release information, and ${L}_{t}$ is tabulated as in Table 4.
- Threshold of inequality.
  (i) The Reservoir Storage Capacity Threshold is as follows, MCM (million cubic meter):
    3 $${6}.{24}\;{\text{MCM}} \le {\text{St}} \le {23}.{44}\;{\text{MCM}}\;\left( {{\text{for}}\;{\text{t}} = {\text{Jan}},\;{\text{Feb}},\; \ldots ,\;{\text{Dec}}} \right)$$
  (ii) The following is the release threshold:
    4 $${3}.{28}\;{\text{MCM}} \le {\text{Rt}} \le {5}.{22}\;{\text{MCM}}\;\left( {{\text{for}}\;{\text{t}} = {\text{Jan}},\;{\text{Feb}},\; \ldots ,\;{\text{Dec}}} \right)$$

#### Penalties

- Penalty functions.
  By penalising the objective function using equation as shown below able to handle the reservoir capacity constraint.
  5 $$penalty\;1 = \left\{ {\begin{array}{*{20}l} 0 \hfill & {if\;S_{t} < S_{{min}} } \hfill \\ {C_{1} (S_{{min}} - S_{t} )^{2} } \hfill & {if\;S_{t} < S_{{min}} } \hfill \\ \end{array} } \right.$$
  6 $$penalty\;2 = \left\{ {\begin{array}{*{20}l} 0 \hfill & {if\;S_{t} < S_{{max}} } \hfill \\ {C_{2} (S_{t} - S_{{max}} )^{2} } \hfill & {if\;S_{t} < S_{{max}} } \hfill \\ \end{array} } \right.$$
  in which ${C}_{1}$ and ${C}_{2}$ are penalty coefficients; ${S}_{t}$ is expressed as storage while ${S}_{min}$ and ${S}_{max}$ are minimum and maximum storage value, respectively.

#### Finalised objective function

The empirically determined values of the two penalty coefficients represent the tolerance of the objective function for its ultimate value, and this has been involved the flood risk reduction as of the storage is the main factor in a reservoir. As a result, Eq. (7) has been used to rewrite the final goal function modification for the optimisation operation rule at the KGD on a monthly basis^18^. In which, *Y* is the fitness recursive objective function.

7 $$Min\;Y = Min\;Z + penalty\;1 + penalty\;2$$

### Proposed MHAs

#### Harris Hawk Optimisation (HHO) Algorithm

Predators with intelligence and distinction, such as the Harris hawk, can hunt, encircle, flush out, and kill prey in groups, such as rabbits. The population of hawks is considered to be a group of hawks that employ seven distinct killing tactics to chase the target rabbit (the answer to the optimisation problem). Initially, the head of the hawks will attempt to catch the prey. If the prey's movement and ability to escape make it impossible for the head of hawks to catch the prey, the group will switch tactics. The remaining members (the hawks) will continue to hunt the prey until it is killed. Hawks are able to hunt the escaping prey by confounding and exhausting it. In the HHO, Harris hawks are classified as candidate solutions, whilst the intended prey is defined as the optimal or global solution. Consequently, the HHO undergoes exploratory and the zigzag motion of the prey during the escaping initiated the Lévy flight distribution character in Harris Hawk with short length jump and boost the searching strategies in exploitative phases^19^. The following are the descriptions of the exploration and exploitation phases of the HHO's^20^.

**Figure 1:**
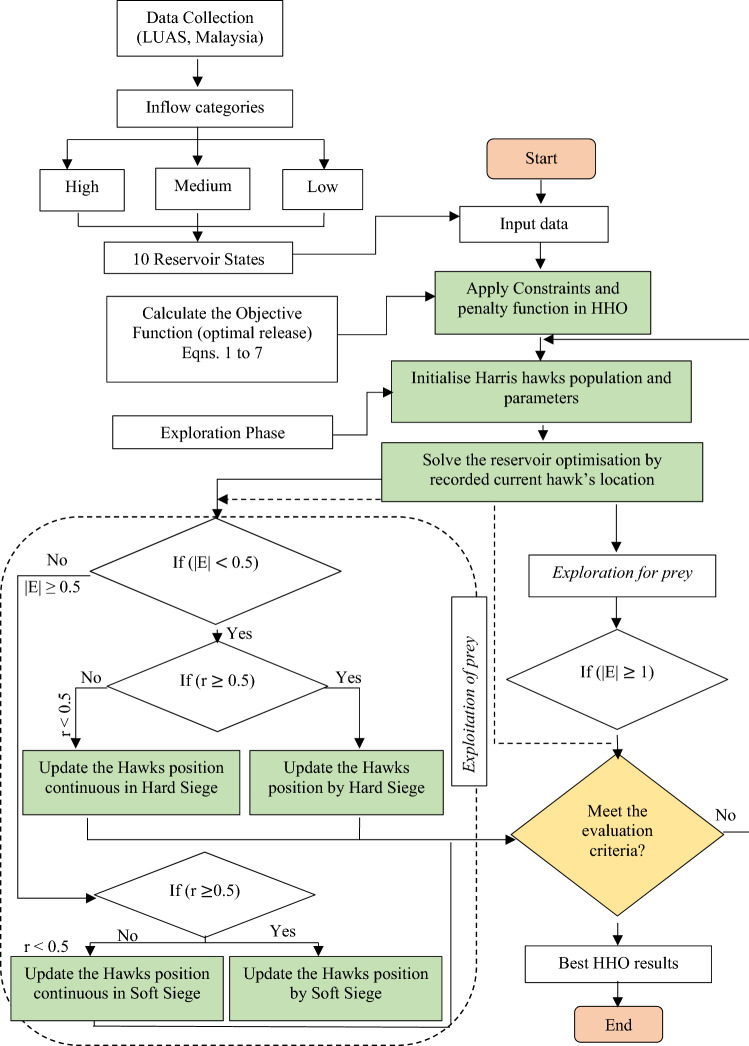
Flow chart of the HHO model executed in year 2001–2019.

Figure 1 depicts the simulation framework for optimising reservoir release curve operations, which began with the collection of data and proceeded to the model algorithm's final process, which was performed with a Harris hawk optimisation (HHO) algorithm. For the years from 2001 to 2019, data was collected at LUAS (Lembaga Urusan Air Selangor) for a total of 19 years (2001–2019). The storage volume initially consisted of ten monthly states, which have since been separated into individual volumes of storage volume. The contrast in the minimum and maximum storage capacities of the reservoir, denoted by S_min_ and S_max_, was determined by using *N* as the state option, and the resulting value was then segmented into *N − 1* intervals. As a result, the discretisation of the storage problem has been resolved and is shown in Table 1. The target storage is the discrete storage values that are used as input data for the simulation and optimisation models. The study begins with examining the accuracy level of the HHO executed during optimal reservoir operation at the KGD. Then, it proceeded with the HHO initialization adjustment of epsilon and dimension of the population. The KGD thresholds were then incorporated into the development of the HHO algorithm to ensure the reservoir operated optimally. Until the ideal HHO strategy was discovered, the HHO algorithm went through various iterations and phases to discover the optimal solution (Hawks has found the rabbit, and this is the candidate solution) as illustrated in Fig. 1.

**Table 1:** Discrete storage, *d* (MCM).

State	Discrete storage (MCM)
1	6.24
2	8.15
3	10.06
4	11.98
5	13.88
6	15.80
7	17.71
8	19.62
9	21.54
10	23.44

#### Opposite-based learning (OBL)

**Figure 2:**
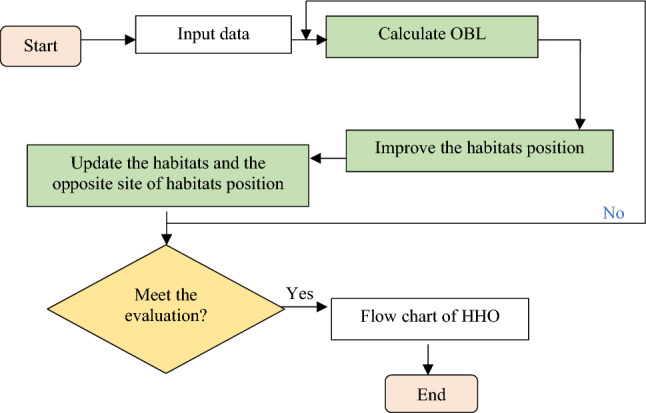
OBL-HHOA.

The Opposite-based learning (OBL) concept was originated by Ref.^21^. The main idea behind the OBL is to search for both an estimate and its opposite estimate at the same time, in order to obtain a better estimate of the present candidate solution. The study was demonstrated by Ref.^22^, who applied the OBL and other integration of the enhancement techniques to solve the real-world that involved medical datasets. The OBL is an effective technique for exploring issue space more thoroughly. By simultaneously searching for the solution and its inverse, this approach can help increase efficiency. While this method increases the computing strain on the algorithm, it however significantly speeds up convergence. The following equation presented the OBL strategy in D-dimensional space. The simplified flow of the OBL for this study's investigation at KGD is represented in Fig. 2.

8 $$\widetilde{x}_{j} = u_{j} + l_{j} - x_{j} \quad j = 1, 2, \ldots ,D$$

where $x$ denotes habitat in search space; $\widetilde{x}$ denotes opposite site of habitat which are generated within the interval of [*u,l*]; $j$ denotes each dimension of the opposite number.

**Figure 3:**
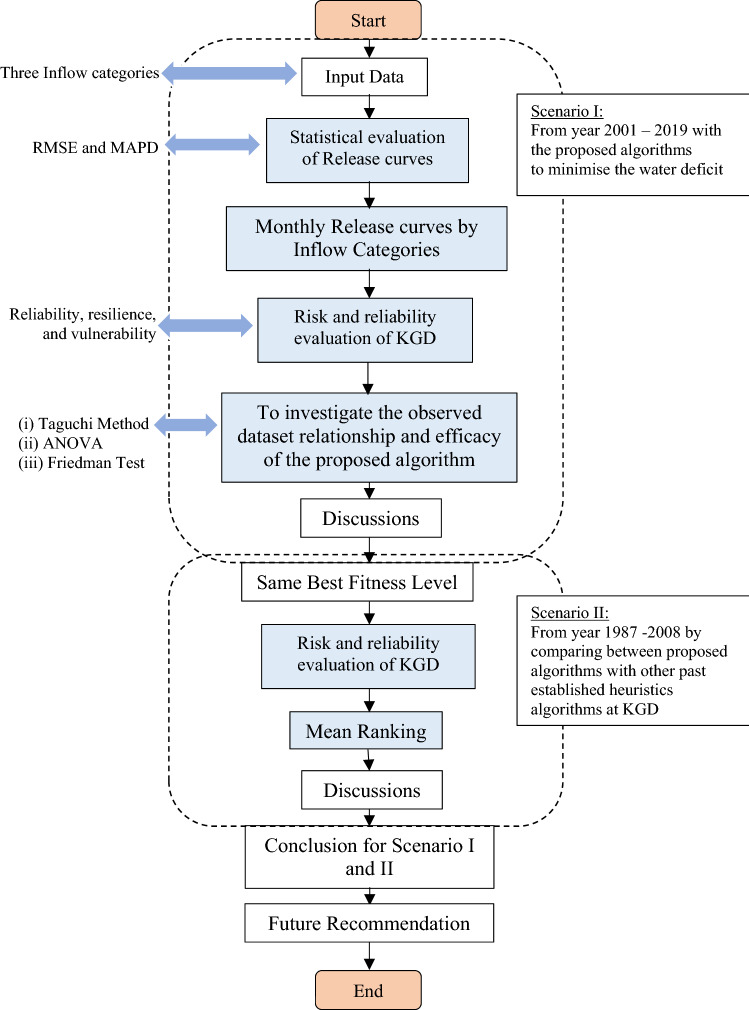
Flow chart of the overall study.

Figure 3 depicts the overall flow chart of the study. It presents two sections of the scenarios, in which in the first scenario, the proposed algorithm optimised the reservoir at the KGD from year 2001 to 2019. The second scenario study compared the established algorithms, namely the ABC, PSO, GA, and the proposed algorithms of this study, the HHO and OBL-HHO from year 1987 to 2008. Following that, the process with the input data continues by segregating the inflow categories into high inflow, medium inflow, and low inflow into the ten different discrete storage, *d* (Table 1) in order to identify the best monthly release curves. The evaluation of the optimisation in the HHO and the OBL-HHO is also divided into two sections: (1) statistical evaluation (root mean squared error, RMSE and mean absolute percentage deviation, MAPD) and (2) risk and reliability evaluation of the HHO and OBL-HHO models. The second scenario implemented the same best accuracy level as in the first scenario, but the investigation, however, spanned the years 1987 to 2008. The purpose of this scenario, reiterated here, is to compare the proposed algorithms to other well-known published heuristic algorithms that were conducted earlier at the KGD, as well as to determine the efficacy of the HHO and OBL-HHO, by assessing the risk and reliability of the optimisation release at the KGD’s operation. The loop of flow chart (see Fig. 3) ends with the study’s conclusion and recommendations. Table 2 tabulated the range of the parameter settings for both HHO and OBL-HHO algorithms.

**Table 2:** Parameter settings for the variable in HHO and OBL-HHO.

Algorithms	Parameter setting
HHO	Population = 10
	Iterations = 500
	Dimensions = 30
	β = 1.5
OBL-HHO	Population = 10
	Iterations = 500
	Dimensions = 30
	Threshold = 2
	CSV = 0.5
	β = 1.5
	a_1 = 10
	a_2 = 0.00005
	a_3 = 0.005
	∂_1 = 0.9
	∂_2 = 0.1

### Performance evaluation of the reservoir system at the KGD

#### Statistical performance evaluation

There are several statistical performance evaluations from Eqs. (9) and (10) below, that were applied to examine and determine the curve that is closest to the targeted demand for this release curves obtained. In general, release curves that are closest to demand will be able to provide the least amount of water deficit. In other words, the least error in both equations resulted in the smallest water deficit.

The root mean squared error (RMSE) is the square root of the mean of all errors squared. The RMSE is widely used, and it is regarded as an excellent general-purpose error metric for statistical evaluation.

9 $$RMSE=\frac{1}{d}\times \sum_{i=1}^{d}\sqrt{{{(x}_{i}-{D}_{t})}^{2}}; \quad t=Jan, Feb, \dots ,Dec$$

Because of its very intuitive interpretation in terms of relative error, the mean absolute percentage deviation (MAPD) is usually expressed the accuracy of the ratio by defined as Eq. (10). It is also commonly used as a loss function in model evaluation.

10 $$MAPD= \frac{1}{d}{\sum }_{t}^{T}\left|\frac{{D}_{t}-{R}_{t}}{{D}_{t}}\right|; \quad t=Jan, Feb, \dots ,Dec$$

where d indicates the number of discrete storage states known to be 10; ${x}_{i}$ denotes release amount obtained from the curve in any storage states *i;*${D}_{t}$ represents targeted demand for any month *t*; ${R}_{t}$ is release for any month *t.*

#### Risk and reliability analysis

For the purpose of calibrating the release policy, simulation was used to conduct a risk analysis on the model. To assess the effectiveness of the optimisation at the KGD, three different indices criteria (reliability, resilience, and vulnerability) were used^23^. The definitions of these three indices in measuring performance levels are mathematically expressed as Eqs. (11)–(14). The periodic ($${R}_{p})$$ is extract from^24^ and incorporated into Eq. (11). Periodic reliability is the most important metric for determining a model’s efficiency in order to meet the goals of a reservoir optimisation model. The higher the $${R}_{p}$$ indices, the more reliable the system.

11 $${\text{Periodic}}\;{\text{Reliability}},\;R_{p} = \left( {\frac{n}{N}} \right) \times 100\%$$

The most important index for checking the model’s performance to meet the goals during a reservoir optimisation process is resilience. The ability of a model to recover from a series of subsequent failures is another definition of resilience. The higher the resilience index, the faster the system recovers during the difficult period.

12 $$Resilience= \frac{NS}{NT}$$

Vulnerability is an expression of the degree to which a model’s failure criteria are met. The lower the vulnerability index, the more robust the system is. The expressed equation is as follows:

13 $$V= \frac{1}{m}\times \sum_{t=1}^{Nv}\left[\text{max}\left(0, {D}_{t}-{R}_{t}\right)\right]\,\, for\,\, t=\text{1,2},\cdots N$$

The shortage index *(SI)* conveys knowledge about both the periodical reliability and severity of the deficit. Equation (14) calculate the shortage index.

14 $$SI= \frac{100}{T} \sum \left(\frac{annual\;deficit}{annual\;demand}\right)2$$

in which $n$ is the total number of time periods meeting the demand; *N* is the total number of considered time period; $NS$ is the the number of satisfied time period followed by a failure (shortage period); $NT$ is the the number of total failure period; m is the no. of model failure period (water deficit ≠ 0); *Nv* is the total time period considered for simulation (in months); *D*_*t*_ is the targeted demand for any month *t*; *R*_*t*_ is the water release for any month *t*.

## Results and discussion

### Scenario I: Applying the HHO Algorithm at the KGD from year 2001 to 2019

#### Statistical evaluation

**Figure 4:**
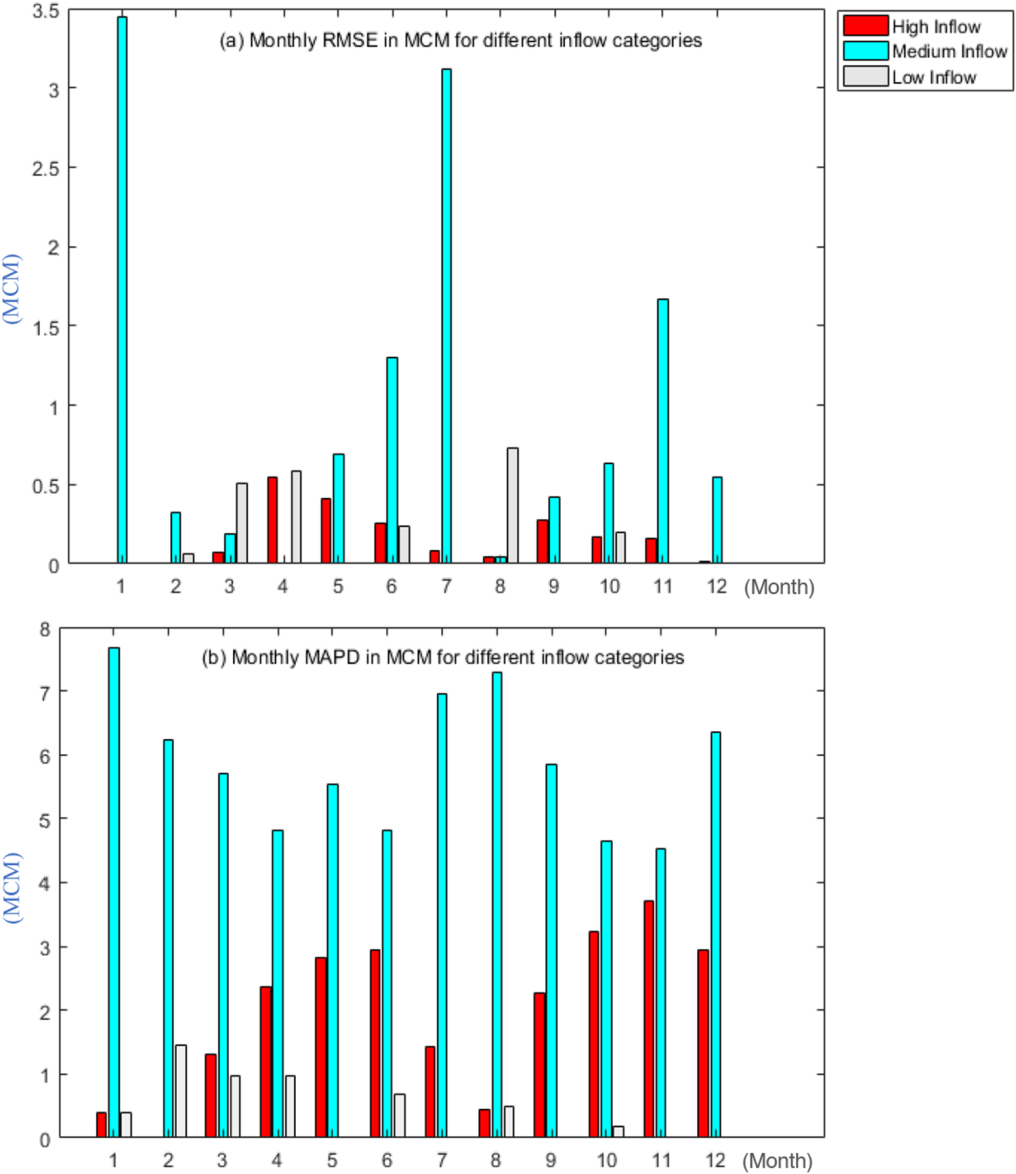
(**a**) and (**b**) Monthly RMSE and MAPD in MCM in different inflow categories.

Figure 4a,b show the statistical evaluations of RMSE and MAPD from 2001 to 2019 (monthly) and classified according to the three distinct inflow categories. Figure 4a shows that the medium inflow category had the highest RMSE (unit MCM) in January, with a value of 3.44, while the high and low inflow categories showed no significant difference. The highest RMSE obtained in the high and low inflow categories occurred in April, with values of 0.54 and 0.59, respectively. The overall RMSE for medium inflow was 12.37MCM, with low inflow at 2.31MCM and high inflow at 2.03MCM.

Figure 4b depicts the MAPD for the monthly release and targeted demand for the HHO algorithm at the KGD. According to Fig. 4b, the medium inflow category provided the highest MAPD for every month of the year, with an average of 5.82MCM. The high inflow category averaged 1.90MCM in November, with the highest MAPD of 3.70MCM. The low inflow category had an average MAPD of 0.47MCM, with the highest MAPD occurring in February at 1.45MCM. The goal of the statistical evaluations is to determine whether the release and targeted demand are capable of reducing the deficit. However, the following risk and reliability evaluations are more important because they explain whether the optimisation systems at the KGD executed from 2001 to 2019 by utilising the HHO algorithm, are capable of achieving the study's objective function.

#### Release curve

**Figure 5:**
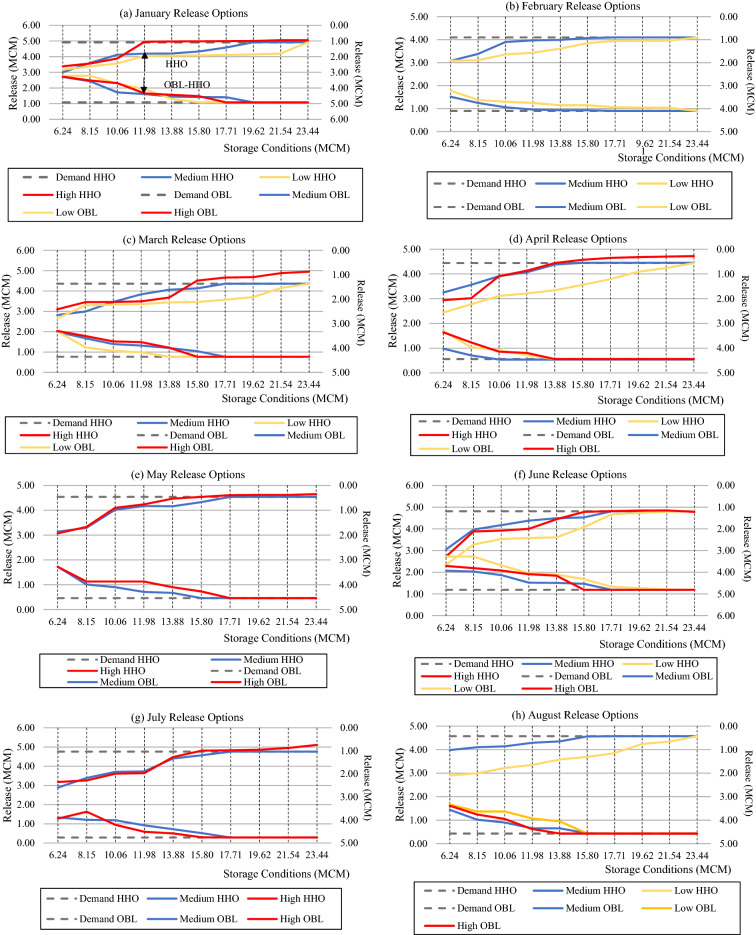

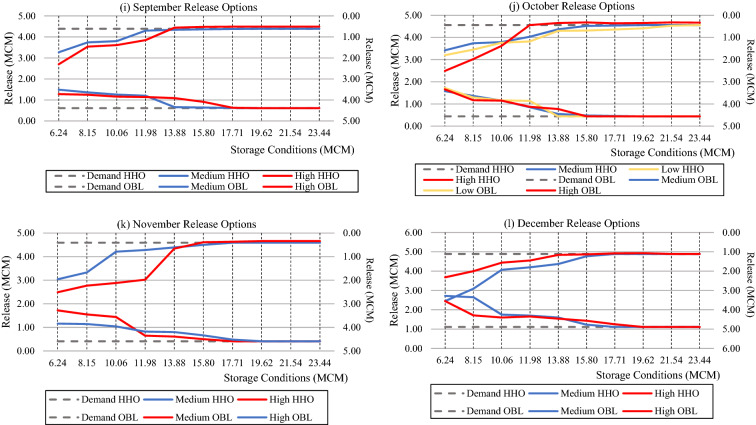
(**a**)–(**l**) Optimal policies of release between year 2001 to 2019 from January to December between HHO and OBL3.1.3Performance Evaluation in Terms of Reliability and Risk Analysis.

The ideal release choice for each storage type was found for each month of the year using the HHO and OBL-HHO algorithms developed, as seen in Fig. 5, from 2001 to 2019. The graphs compare initial reservoir storage to reservoir monthly release per unit of demand for the high, medium, and low flow conditions. The primary goal is to generate a release curve that depicts the best release policy in response to changes in inflow. The figures depict the optimal reservoir storage volume and release rates, with the goal of minimising expected device output.

Figure 5a–l demonstrates the release curves for graphs with a normal Y-axis (Left side and upper line graphs) which were generated using the HHO technique, whereas graphs with a reversed Y-axis (reversed scale) (Right side and lower line graphs) were generated using the OBL-HHO algorithm. The months of the individual release curves are depicted below. Beginning with Fig. 5a for January, the HHO was the first high inflow category curve to satisfy demand with 4.92 MCM at 11.98 MCM storage level, while the OBL-HHO met demand at 17.71 MCM. Although the HHO met demand before the OBL-HHO, there was a 0.13 MCM water loss near the end of storage. The OBL-HHO achieved close proximity to the demand line at 10.06 MCM initial storage with 4.28 MCM, whereas the HHO only achieved 4.12 MCM. Both models met the demand threshold at 19.62 MCM storage level. For the lower inflow category, the OBL-HHO could fulfil the demand line at 15.80 MCM storage level, whereas the HHO could only do so at 23.44 MCM. Figure 5b depicts only medium and low inflow categories for February, and the OBL-HHO meets the 4.10 MCM demand line faster than HHO.

Figure 5c,d,f,h,j illustrate the scenarios within the three inflow categories. For March, June, and October, the graphs showed that the OBL satisfied the demand line at 15.80 MCM without wastage. Both methods generated identical results for the high inflow between April and August at 13.88 MCM storage level. Nonetheless, it reveals that the HHO algorithm during the high inflow category tends to waste between 2.4 and 13.5% of water, with March and October being the months with the utmost water loss, whereas in the medium and low inflow categories, both algorithms provide comparable stability with no water loss.

Figure 5e,g,k,l only illustrate high and medium inflow (l). In May and December, the HHO could provide downstream demand with 15.80 MCM of high inflow storage. The OBL-HHO was able to satisfy the demand line one stage later than the HHO at 17.71 MCM and 19.62 MCM for high inflow in May and December, respectively. Both algorithms worked effectively with no waste in medium inflow, and the OBL-HHO met demand at storage level of 15.80 MCM in May and 17.71 MCM in December. No waste scenario happened in July for high and medium inflows using the model OBL-HHO. Finally, it come to the months of September and November, respectively. Both algorithms hit the medium inflow demand line at storage level of 19.62 MCM and 17.71 MCM for both months. For high inflow, the OBL-HHO satisfied demand at storage level of 19.62 MCM with no water loss.

Table 3 presents the periodic reliability performance between the HHO and OBL-HHO. A total of 228 results from the year 2001 to 2019 were obtained from the HHO and OBL-HHOs optimisation models, with the three different inflow categories. There is no shortage period reported for the high inflow category for both optimisation models as the inflow is sufficient to supply according to the demand. However, the surplus period (exceeding water demand) occurred 21 times (9.21%) for the HHO and 28 times (12.28%) more frequently for the OBL-HHO, respectively. During the period of high inflows, it was observed that the HHO achieved 30 times (13.16%) and the OBL-HHO achieved 21 times (9.21%) by meeting the demand. For the medium inflow category, the HHO was able to achieve 88 times (38.60%) of the exact demand, whereas the OBL-HHO achieved 47 times (20.61%), 1.87 times less than HHO. During the surplus period, HHO and OBL-HHO produced 35 times (15.35%) and 62 times (27.19%), respectively. During the shortage period with medium inflow, HHO was delivered 38 times (16.67%) and OBL-HHO was obtained 54 times (23.68%). For the shortage period in low inflow categories, the HHO and OBL-HHO achieved consistent outcomes with 9 times (3.95%) and 10 times (4.39%), respectively. The results obtained to meet the exact demand for low inflow categories, for the HHO and OBL-HHO, were 7 times (3.07%) and 6 times (2.63%), respectively.

**Table 3:** Periodic reliability performance of the HHO model from the year 2001–2019.

Inflow categories	Surplus period	Exact period	Shortage period
HHO			
High	21 times (9.21%)	30 times (13.16%)	0 time (0%)
Medium	35 times (15.35%)	88 times (38.60%)	38 times (16.67%)
Low	0 time (0%)	7 times (3.07%)	9 times (3.95%)
Total no. of release	56 times (24.56%)	125 times (54.83%)	47 times (20.61%)
OBL-HHO			
High	28 times (12.28%)	21 times (9.21%)	0 time (0%)
Medium	62 times (27.19%)	47 times (20.61%)	54 times (23.68%)
Low	0 times (0%)	6 times (2.63%)	10 times (4.39%)
Total no. of release	49 times (21.49%)	163 times (71.49%)	16 times (7.02%)

**Figure 6:**
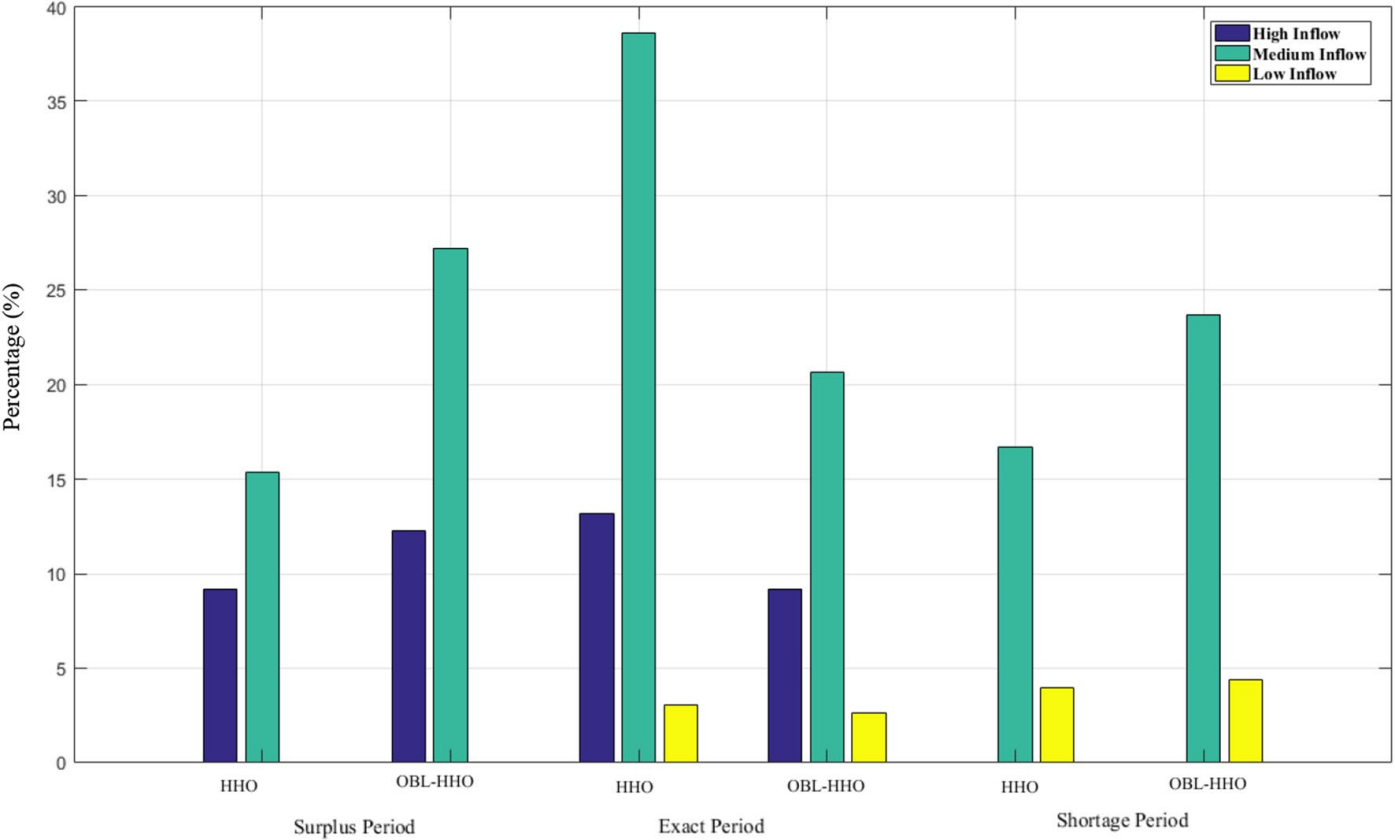
Periodic Reliability Performance in percentage classified according to inflow categories.

Figure 6 illustrates the percentage of periodic reliability between the HHO and OBL-HHO. Based on the observations reported in Fig. 6 and Table 2, the overall findings demonstrate that the HHO yielded significantly superior reliability across all three inflow categories. Throughout the end, without considering the inflow categories and solely comparing the efficacy of HHO and OBL-HHO, the average percentage of reliability demonstrated by the HHO during the surplus period was 8.19%, but the OBL-HHO achieved 13.16%, which was 4.97 percent higher than the HHO algorithms. During the exact demand, the average percentage of reliability attained by the HHO was 18.28%, whereas the OBL-HHO achieved only 10.82%. The HHO remains superior to OBL-HHO in terms of the average percentage of reliability encountered during shortage periods, with 7% versus 9%, respectively. However, as shown in Table 3, there are other reservoir evaluation risk analyses.

Table 4 depicts the performance evaluation for the HHO and OBL-HHO models at the KGD. The model's reliability refers to how often the model achieves success and failure, which indicates how dependable it is. Table 3 and Fig. 6 demonstrate the wastage and meeting demand percentages. According to the shortage index evaluation criteria, there is no significant difference between the high and low inflow categories for the HHO, whilst for the OBL-HHO, the shortage index for high and low inflow attained 0.00008 and 0.00004, respectively. However, it obtained the highest shortage index for the medium inflow category for both the HHO and OBL-HHO, indicating that the system may face a water shortage in the future. As a result, it is necessary to determine how well the system will recover from the failure. The next evaluation is resiliency; it can be seen that for the HHO model, it was capable of recovering from failure during medium and low inflow events; however, during high inflow events, the model was unable to recover and may breach the storage constraint, whereby the condition for the OBL-HHO in resiliency was similar to the HHO. Thus, we may need to be more aware of this incident, which we do not want to occur. Finally, the vulnerability index demonstrated the least value in a more robust manner. Thus, the least value sequence begins with medium, high, and low inflows of 0.34, 0.63, and 1.23, respectively, whereas for the OBL-HHO attained 0.20, 0.49, and 0.96, respectively for medium, high, and low inflow categories. In this vulnerability evaluation reservoir risk analysis, however, there is a slightly significant difference between the OBL-HHO and HHO, with the OBL-HHO model being slightly more robust than the HHO for all three inflow categories. In terms of periodic reliability performance, the HHO model is superior to the OBL-HHO model, and the system is more competent to "bounce back" from a failure during the period of analysis from 2001 to 2019.

**Table 4:** Performance evaluation of the HHO model from the year 2001–2019 (monthly).

Algorithms	Evaluation/inflow categories	High	Medium	Low
HHO	Wastage due to excess release (%)	9.21	15.35	0
	Meeting demand (%)	13.16	38.60	3.07
	Shortage index	0.00007	0.00024	0.00005
	Vulnerability	0.63	0.34	1.23
	Resiliency	–	3.24	0.78
OBL-HHO	Wastage due to excess release (%)	12.28	27.19	0
	Meeting demand (%)	9.21	20.61	2.63
	Shortage index	0.00008	0.000021	0.00004
	Vulnerability	0.49	0.20	0.96
	Resiliency	–	2.02	0.60

#### Response graph of parameters with the HHO model

**Figure 7:**
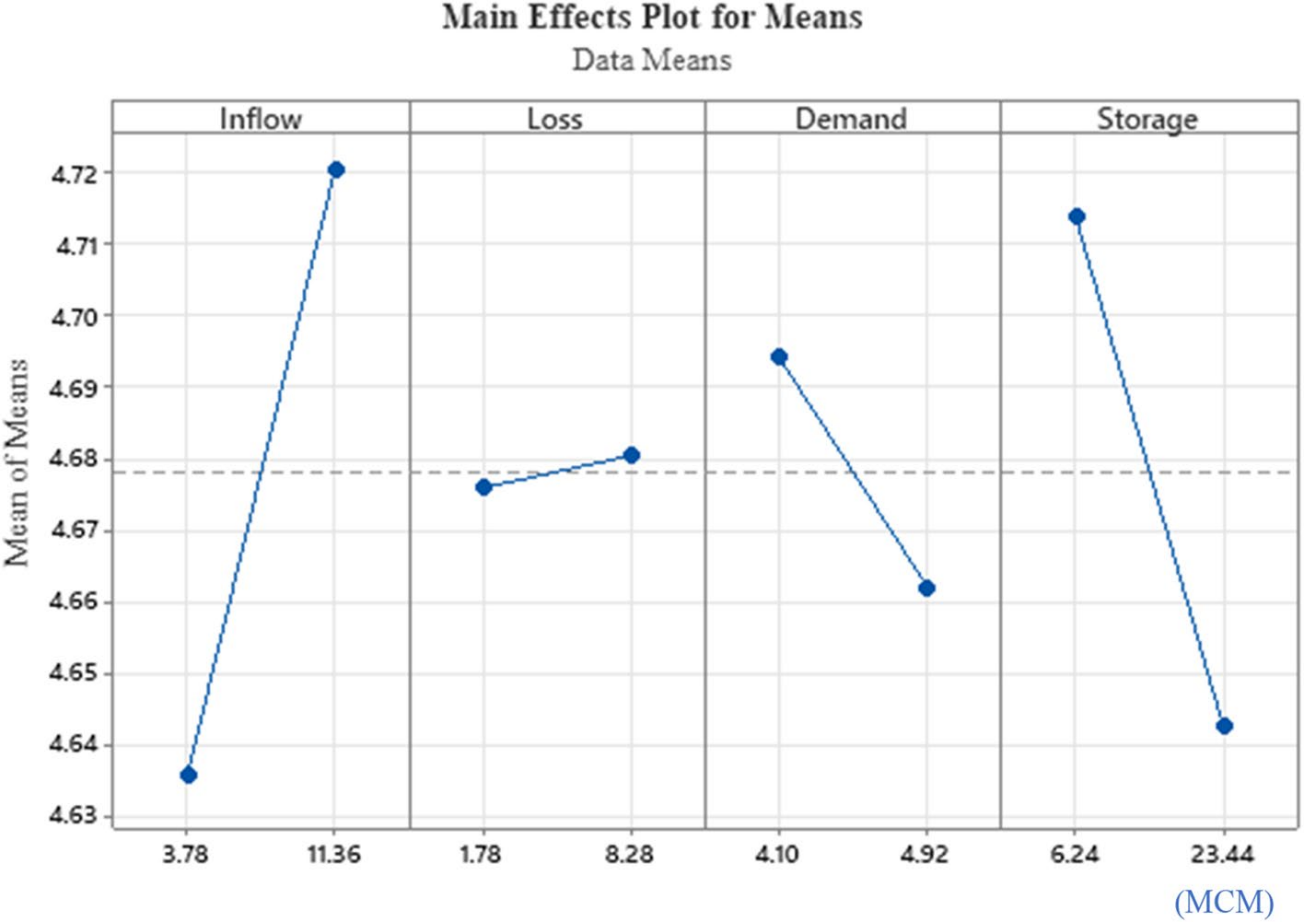
Graph of response for the HHO model.

Using the Taguchi model, the optimal parameters for the algorithms can be determined efficiently^25^. The Taguchi technique uses orthogonal arrays to calculate the minimum number of experiments required to get all of the factors impacting the output parameter. The orthogonal array performed in this subsection of L16 (2^4^) is aimed at exploring the influence of 4 factors with 2 levels of values consisting of an upper and lower boundary in 16 experiment runs. The primary effect plot for the simulated releases is depicted in Fig. 7. Inflow had the greatest effect on the simulated release at KGD, followed by storage, demand, and loss. The factor is insignificant in the response graph if the line depicted is horizontal because there is no change in response to the factor. However, if the line graph shows a steep slope, this indicates that the factor significantly affects the response.

A univariate ANOVA was used to investigate the impact of factors on the dependent variables using the measured data from the Taguchi method's analysis of mean variance. The findings of the univariate ANOVA for the impact of the various factors on the independent variables and the interactions between the factors by one to one are depicted in Table 5. SS and MS represent the sum of square and mean square, respectively. The effects by each factor on the independent variables were identified as in statistically significant (p < 0.05), while the percentage of the contribution (PC), were then verified as in previous response Table 5 , showed that inflow factor was the main effect as this could be proved in Table 5 as the PC obtained was the most contributed with 11.55%, thus it is rank in first for the main effects plot. Whilst, for the interaction between the factors, the most PC obtained was demand*storage, 32.07%, followed by the 19.72% and 12.10% of inflow*demand, and inflow*storage, respectively.

**Table 5:** ANOVA table.

Source	DF	SS	MS	F-value	P-Value	PC (%)
Inflow	1	0.028730	0.028730	5.40	0.068	11.55
Loss	1	0.000081	0.000081	0.02	0.907	0.03
Demand	1	0.004160	0.004160	0.78	0.417	1.67
Storage	1	0.020306	0.020306	3.81	0.108	8.16
Inflow*Loss	1	0.000000	0.000000	0.00	1.000	0.00
Inflow*Demand	1	0.049062	0.049062	9.22	0.029	19.72
Inflow*Storage	1	0.030102	0.030102	5.66	0.063	12.10
Loss*Demand	1	0.006241	0.006241	1.17	0.328	2.51
Loss*Storage	1	0.003721	0.003721	0.70	0.441	1.50
Demand*Storage	1	0.079806	0.079806	14.99	0.012	32.07
Total	15	0.248826	0.248826			100

#### Ranking analysis—Friedman test

Numerous non-parametric tests, such as the Sign test and the Wilcoxon-Signed-ranks test, can be employed to handle pairwise comparisons. However, in this study, the Friedman test is used to compare the outcomes of the evaluation of the suggested algorithms' exploration and exploitation capabilities, respectively. The Friedman test was chosen since it was used to compare more than two algorithms. If there is statistically significant difference, hence, post-hoc methods such as the Bonferroni-Dunn procedure will be applied. This can be checked by adopting the following equations for Friedman test and Chi-square, respectively as below:

15 $$E\left(R\right)= \frac{N\left(k+1\right)}{2}$$

16 $${X}^{2}= \frac{12}{N\cdot k\cdot (k+1)}\cdot \sum {R}^{2}-3\cdot N\cdot (k+1)$$

in which *N* is the total number of rows; *k* is the total number of columns; ${R}^{2}$ is the sum of the ranks, and ${X}^{2}$ is the Chi-square.

According to Friedman's average rankings, the most efficient algorithm obtained is the HHO. The statistically significant difference was determined using the Chi-Square distribution table, *d*_*f*_ was calculated as the degree of freedom by subtracting the *k − 1*, which was equal to 2. Chi-square values of *X*^*2*^ = 5.3 and − 6.3 were obtained. The critical Chi-Square value obtained from the Chi-Square distribution table is 5.991 (0.05, and *d*_*f*_ = 2). Both *X*^*2*^ values obtained fell below the critical Chi-Square distribution table, indicating that there is no statistically significant difference. As a result, no post-hoc analysis was needed.

### Scenario II: Comparing the HHO Algorithm and OBL-HHO with other published heuristic algorithms at the KGD from the year 1987 to 2008

The total of 22-year rainfall data beginning from year 1987 to 2008 were obtained from an investigation conducted by Ref.^26^. Then, in the study, the authors had utilised meta-heuristic algorithms, namely the Artificial Bee Colony (ABC), Particle Swarm Optimisation (PSO), and Genetic Algorithm (GA) carried out at the KGD. This section of our analysis compares their findings to our suggested HHO and OBL-HHO algorithms, using the similar duration and method of inflow state calculation as found in Ref.^26^. In this section, the performance indices are measured using million gallons (MG) as the unit of measurement. As explained in “Performance evaluation of the reservoir system at the KGD”, the equations for determining the optimal reservoir release remain unchanged. However, in order to calculate the losses shown in Table 4, they must be converted into units of MG to commensurate with those of the previous study. The inflow states have been conducted by Ref.^27^ to convert the amount of rain during that time period into inflow are explained in the following equation. In his study, the 50% of the total rainfall amount goes into the KGD’s catchment area.

17 $$I_{t} = 0.5 \times catchment\;area \times rainfall_{t}$$

where *I* is the inflow of the month *t.*

**Figure 8:**
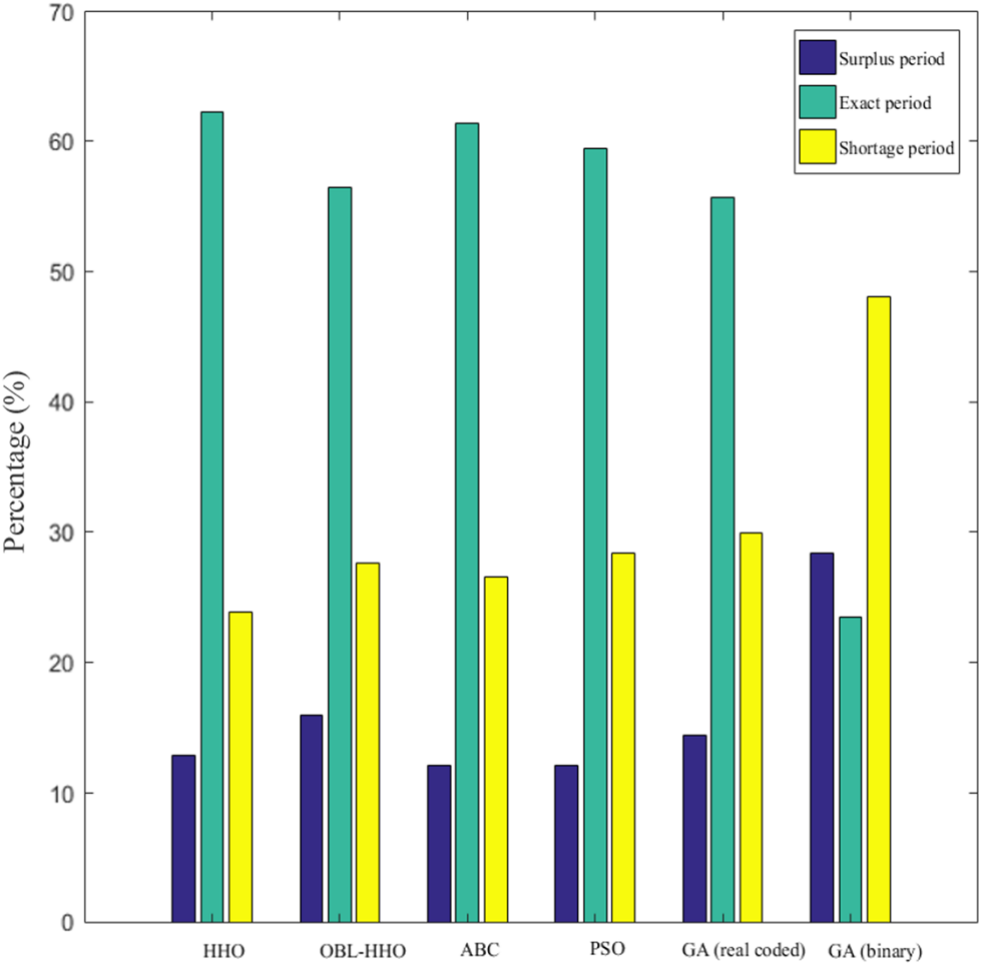
Monthly periodic reliability from year 1987 to 2008.

**Figure 9:**
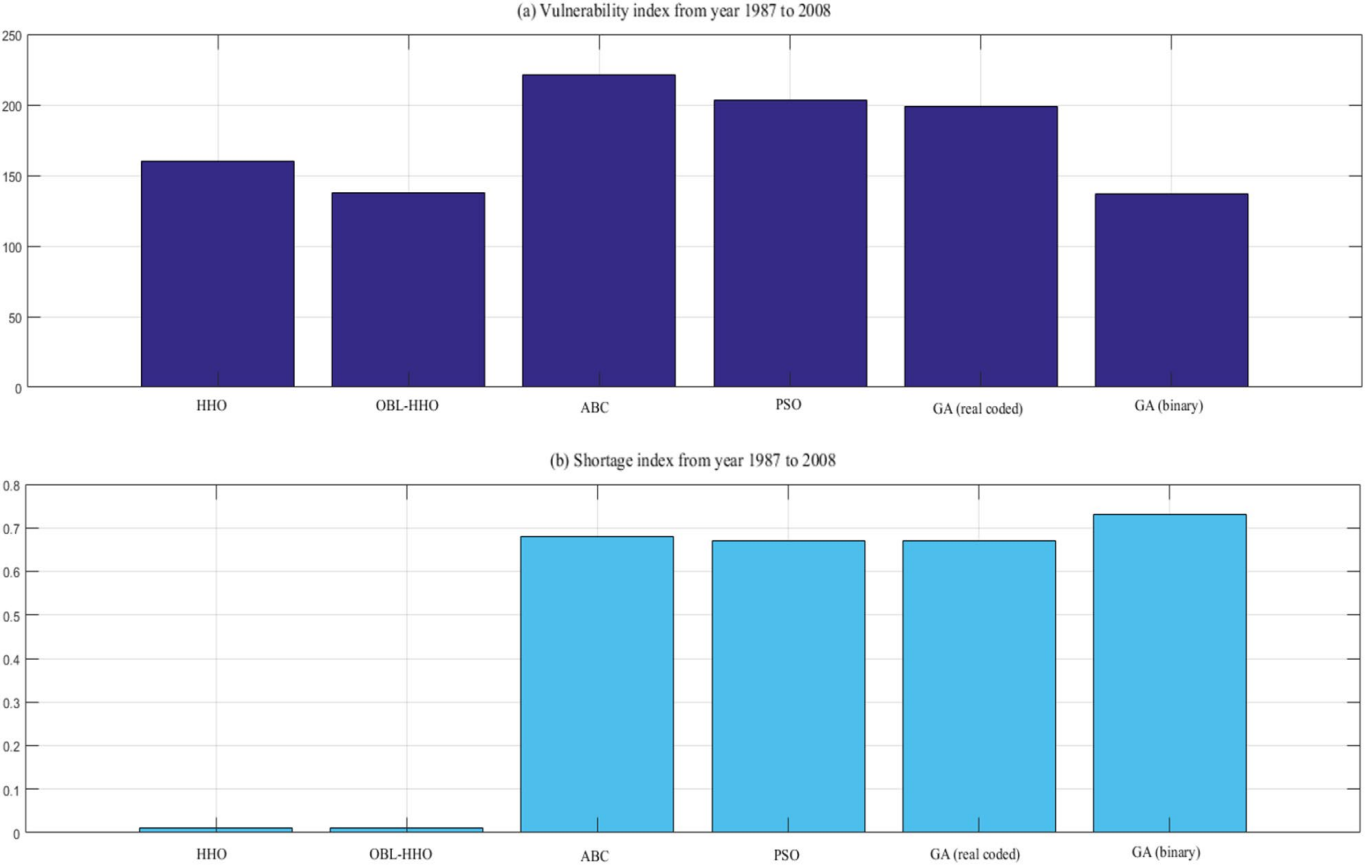
(**a**) Vulnerability index and (**b**) shortage index from year 1987 to 2008.

**Figure 10:**
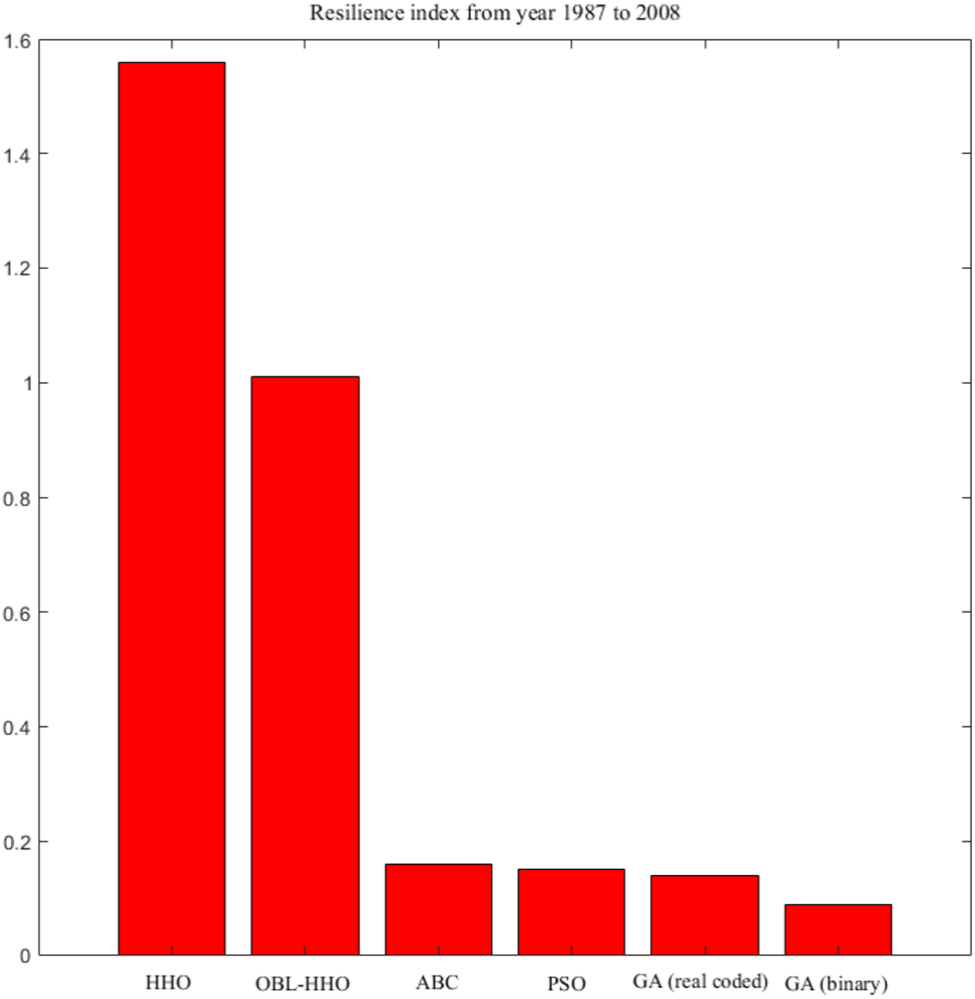
Resilience index from year 1987 to 2008.

The benchmarking to the category of the inflow is the same as earlier Scenario I. The findings of the performance in reliability and risk analysis for HHO, ABC, PSO, and GA are represented from Figs. 8, 9 and 10, respectively. The mean ranking *(MR)* for the overall MHAs is presented in the final section of this Scenario II to provide a better illustration of the performance evaluation index of the algorithms^28^.

18 $$MR= \frac{1}{n}\sum_{i=1}^{n}{rank}_{i}$$

where *n* is the number of evaluation index in Scenario II, *n* = 4.

#### Comparative assessment of Reliability Indices from 1987 to 2008

The overall performance of several meta-heuristic algorithms at the KGD is depicted in Fig. 8. The entire investigation duration was 264 months, and the results demonstrated that the HHO and OBL-HHO algorithms met demand (exact period) with a 63.26% and 56.44% reliability, respectively. According to Ref.^26^, the ABC outcomes satisfied demand in 61.36%, 59.47% in PSO, 55.68% in real-coded GA and 23.5% in binary GA. For the excess release (surplus period), which resulted in wastage, the HHO achieved 12.88%, OBL-HHO exceeded with 15.91%, while ABC and PSO both attained similar reliability of 12.1%. However, the GA in binary generated the most wastage at 28.4%, while the GA in real-coded form generated a 14.4%. The sequence for the optimal release policy's shortage period started with the worst placement, where the binary GA, real-coded GA, PSO, OBL-HHO, ABC, and finally the HHO with the percentage of 48.10%, 29.92%, 28.41%, 27.65%, 26.54% and 23.86%, respectively.

#### Comparative assessment of vulnerability indices from 1987 to 2008

The vulnerability index is depicted in Fig. 9a from 1987 to 2008. The model's robustness was demonstrated by the lowest vulnerability index. However, the proposed algorithm, the HHO, only obtained the second-lowest vulnerability index value of 160.10. The Binary-GA was the algorithm that gave the least vulnerability index available, with a score of 136.91. In addition, there was no significant difference between the Binary-GA and the OBL-HHO vulnerability index, which attained 137.83. The highest index was that given by the ABC, which had a value of 220.94, while the PSO and real coded-GA had values of 203.27 and 199.15, respectively.

Figure 9b displays the results of the shortage index; with the least index given by the HHO and OBL-HHO at a value of 0.01, respectively. The ABC, PSO and real coded-GA does not show any significant difference amongst themselves, with the value attained 0.68, 0.67, and 0.67, respectively. However, the highest possible of system failure at period was the binary-GA with a value of 0.73. Hence, the following evaluation of resilience is essential to understand how fast the algorithms recover from the failure.

#### Comparative assessment of resilience Indices from 1987 to 2008

Figure 10 shows that the HHO algorithm has the greatest ability to recover from system failure, with a value of 1.56. The next best was the OBL-HHO followed by the ABC algorithm with a value of 1.01 and 0.16, respectively. Then the PSO and real coded -GA, followed with values of 0.15 and 0.14, respectively. The Binary-GA with 0.09 has been the weakest algorithm to recover from the system failure.

Overall, in scenario II, the HHO algorithm had performed well in terms of reliability, shortage index, and resilience. However, HHO failed to meet the vulnerability index criteria. Despite this, the HHO was able to present the resilience index by demonstrating that it is the most capable algorithm among other MHAs for recovering from failure. The binary-GA demonstrated the best performance in vulnerability index criteria; however, the other evaluation indexes demonstrated poor performance, particularly in reliability and resilience. According to Table 6, the overall mean ranking of the algorithms in the evaluation indices revealed that the HHO algorithm is the best among the other MHAs, by obtaining the overall mean ranking of 1.50. Furthermore, the results obtained, particularly the reliability criteria, demonstrated that HHO is capable of minimising the water deficit at the KGD.

**Table 6:** Ranking system in evaluation of risk and reliability analysis.

MHAs/mean ranking for evaluation index	Reliability	Vulnerability	Shortage index	Resilience	Overall mean ranking
HHO	1	3	1	1	**1.50**
OBL-HHO	4	2	1	2	2.25
ABC	2	6	3	3	3.5
PSO	3	5	2	4	3.5
GA (real coded)	5	4	2	5	4
GA (binary)	6	1	4	6	4.25

Significant values are in [bold].

## Conclusions

This study was pursued with the aim of determining the optimal release operations at the KGD using the HHO and OBL-HHO algorithms, to minimise the water deficit. The overall findings for Scenario I demonstrated the HHO algorithm met 38.60% of demand for medium inflows. High inflows could meet 13.16% of demand, and low inflows, 3.07% of demand. With the OBL-HHO, the demand for medium inflow was met at 20.61%, then for high inflow at 9.21%, and for low inflow at 2.63%. The HHO scored 0.34 for medium inflow, 0.63 for high, and 1.23 for low. The OBL-HHO scored 0.20, 0.49, and 0.96 for medium, high, and low inflows, respectively. The final session of the evaluation used a response graph to determine how significantly a variable affects the reservoir release operation at the KGD. For Scenario II, monthly release curves from 1987 to 2008 were used (the monthly release curves for this period were chosen because the aim of this second part was to evaluate the optimal release reservoir operation in terms of reservoir risk analysis performance between the proposed algorithms (our study) and other heuristic algorithms, such as the ABC, PSO, and GA (reported in an earlier study by Hossain (2013)). The HHO performed 62.26%, ABC 61.36%, PSO 59.47%, the OBL-HHO 56.44%, real-coded GA 55.68%, and binary GA 23.5% in reliability performance. In terms of vulnerability indices (in MG), the binary GA scored 136.91, the OBL-HHO scored 137.83, and the HHO scored 160.10. The HHO approach used in the optimal release reservoir operation recovered from a failure better than the OBL-HHO, gaining 1.56. Thus, HHO performed higher in reliability, resilience, and scarcity index.

Based on the results of both case studies, the HHO algorithm is not the overall best algorithm among the other MHAs tested in this study, if all the parameters are considered together at once. Nevertheless, the HHO remains a convincing algorithm for developing monthly release curves, as evidenced by the convincing results obtained in reliability and resilience index, whereas the OBL-HHO is capable of minimising the water deficit at the KGD for the respective monthly release curves during the end storage level phase. Hence, it is up to the reservoir stakeholder to decide the algorithm best correlate to the respective climate condition. In a nutshell, future research will likely examine the impact of climate change on KGD reservoir release operations under various climate scenarios.

## Data Availability

Data and material will be made available on reasonable request to the corresponding author.
